# Prevalence and analysis of *Pseudomonas aeruginosa *in chinchillas

**DOI:** 10.1186/1746-6148-6-52

**Published:** 2010-11-17

**Authors:** Yasuko Hirakawa, Hiraku Sasaki, Eiichi Kawamoto, Hiroki Ishikawa, Tetsuya Matsumoto, Naoki Aoyama, Koh Kawasumi, Hiromi Amao

**Affiliations:** 1 Laboratory of Experimental Animal Science, Nippon Veterinary and Life Science University, 1-7-1 Musashino-shi, Tokyo, Japan; 2 Animal Research Center, Tokyo Medical University, 6-1-1 Shinjuku-ku, Tokyo, Japan; 3 Department of Microbiology, Tokyo Medical University, 6-1-1 Shinjuku-ku, Tokyo, Japan

## Abstract

**Background:**

Chinchillas (*Chinchilla laniger*) are popular as pets and are often used as laboratory animals for various studies. *Pseudomonas aeruginosa *is a major infectious agent that causes otitis media, pneumonia, septicaemia enteritis, and sudden death in chinchillas. This bacterium is also a leading cause of nosocomial infections in humans. To prevent propagation of *P. aeruginosa *infection among humans and animals, detailed characteristics of the isolates, including antibiotic susceptibility and genetic features, are needed. In this study, we surveyed *P. aeruginosa *distribution in chinchillas bred as pets or laboratory animals. We also characterized the isolates from these chinchillas by testing for antibiotic susceptibility and by gene analysis.

**Results:**

*P. aeruginosa *was isolated from 41.8% of the 67 chinchillas included in the study. Slide agglutination and pulsed-field gel electrophoresis discriminated 5 serotypes and 7 unique patterns, respectively. For the antibiotic susceptibility test, 40.9% of isolates were susceptible to gentamicin, 77.3% to ciprofloxacin, 77.3% to imipenem, and 72.7% to ceftazidime. DNA analyses confirmed that none of the isolates contained the gene encoding extended-spectrum β-lactamases; however, 2 of the total 23 isolates were found to have a gene similar to the *pilL *gene that has been identified in the pathogenicity island of a clinical isolate of *P. aeruginosa*.

**Conclusions:**

*P. aeruginosa *is widely spread in chinchillas, including strains with reduced susceptibility to the antibiotics and highly virulent strains. The periodic monitoring should be performed to help prevent the propagation of this pathogen and reduce the risk of infection from chinchillas to humans.

## Background

Chinchillas (*Chinchilla laniger*) are in the Rodentia order, Hystricognathi suborder and the Chinchillidae family. Since chinchillas are small and easy to breed, they are popular as pets. Chinchillas have a large tympanum and cochlear duct similar to the human organ; therefore, they are also often used as laboratory animals for otolaryngological studies [1-4].

*Pseudomonas aeruginosa *is a Gram-negative rod-shaped bacterium and an opportunistic pathogen that causes various serious diseases in humans and animals. Recently, this pathogen has acquired resistance to many therapeutic antibiotics and, in particular, emergence of *P. aeruginosa *isolates with extended-spectrum β-lactamases (ESBLs) has become a serious problem in laboratories and hospitals [5-8]. Thus, it is important not only to investigate the occurrence of *P. aeruginosa *infection but also to assess the existence of antibiotic-resistant *P. aeruginosa *strains to ensure the health of humans and animals. It is known that chinchillas are sensitive to *P. aeruginosa *infection and are therefore frequently used as an animal model of diseases caused by *P. aeruginosa*, including otitis media, otitis interna, pneumonia, septicaemia enteritis, and sudden death [9-12]. In early studies, various clinical treatments against *P. aeruginosa *infection were discovered using chinchillas as an animal model [11,13].

The clinical signs caused by natural infection of *P. aeruginosa *in chinchillas bred as pets or laboratory animals have been described. Doerning et al. [10] reported that the occurrence of *P. aeruginosa *infection in laboratory chinchilla colonies was thought to be transmitted by laboratory equipment such as water and water bottles. The clinical signs of *P. aeruginosa *infection have been shown to include otitis media and otitis interna with neurologic manifestations, as well as fatal symptoms in several animals [14]. Although experimental models of *P. aeruginosa *infection in chinchillas have been well described in recent years, there are only few reports on the distribution and abundance of natural infection in chinchillas that are bred as laboratory animals or pets. Furthermore, in these case reports, minimal epidemiological information was supplied.

Typing of *P. aeruginosa *strains and determining the level of susceptibility to antibiotics are epidemiologically useful for measuring the effectiveness of infection control in chinchillas. Minimum inhibitory concentrations (MICs) are helpful indicators to select therapeutic substances and to detect the effects of previously used antibiotics. Zoonoses have long been considered one of the most important threats in public health [15]. To prevent propagation of *P. aeruginosa *infection among humans and animals, detailed characteristics of the isolates, including antibiotic susceptibility and genetic features, may be needed. It is necessary to investigate this epidemiological relationship not only for the health of laboratory animals and pets but also for human health.

In this study, we surveyed the occurrence of *P. aeruginosa *infection among chinchillas bred as laboratory animals or pets. Subsequently, the epidemiological characteristics of *P. aeruginosa *isolates, including serotype and molecular typing, were determined and we tested for the presence of ESBLs in *P. aeruginosa *isolates by DNA analysis.

## Results

### Surveillance of *P. aeruginosa *infection in chinchillas

A survey was carried out in 67 healthy chinchillas bred both as pets and laboratory animals. Of these animals, 7 of 23 chinchillas bred as pets and 21 of 44 chinchillas bred as laboratory animals were confirmed to be carriers of *P. aeruginosa*. In all, 41.8% of otherwise-healthy chinchillas were found to be carriers of *P. aeruginosa*. The isolation of *P. aeruginosa *was carried out with swabs from the oral cavity, faeces, and water bottles in the chinchilla cage, with the detection rates of those swabs at approximately 7%, 9%, and 20%, respectively. The detection rate of swabs from water bottles showed highly percentage among them. In 2 chinchillas, *P. aeruginosa *was isolated with swabs from the oral cavity, faeces, and water bottles, and in one chinchilla, *P. aeruginosa *was isolated from both oral cavity and faeces.

### Typing of *P. aeruginosa *isolates

**Figure 1:**
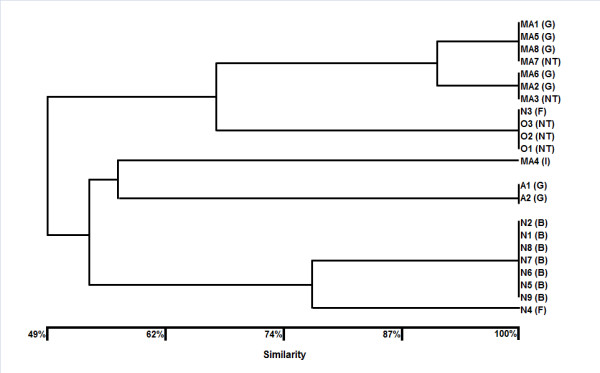
**Dendrogram of a total of 22 strains identified as *Pseudomonas aeruginosa *isolated from chinchillas by the unweighted-pair group method with arithmetic averages based on pulsed-field gel electrophoresis analysis**. The letters in parenthesis indicate the serotype determined by the slide agglutination test kit (Denka Seiken, Tokyo, Japan) as follows: B, serotype B; F, serotype F; G, serotype G; I, serotype I; NT, nontypeable strain.

Figure 1 shows the dendrogram and results obtained from both serotype and pulsed-field gel electrophoresis (PFGE) analysis. The serotype of all *P. aeruginosa *isolates was determined using a slide agglutination test kit. A total of 28 isolates were divided into 4 serotypes (number of isolates): G type (9), B type (11), I type (1), F type (2) and untypeable isolates (5). In the isolates from chinchillas bred as pets, 2 G type and 5 untypeable strains were identified; 11 and 7 B type and G type isolates, respectively, were found in the isolates from chinchillas bred as laboratory animals.

A total of 22 isolates were used to conduct the PFGE analysis because 6 isolates could not be revived after storage. The dendrogram was constructed from the results of the band patterns of the PFGE analysis (Figure 1). Seven unique patterns were observed in *P. aeruginosa *isolates from chinchillas (Additional file 1) and the dendrogram corresponded to these serotypes. The isolates were defined as identical PFGE types if they were 100% identical. Isolates from the same facility were closely related to each other, indicating that the typing of the isolates by PFGE analysis and serotype contributed to epidemiological data.

### Antibiotic susceptibility

**Figure 2:**
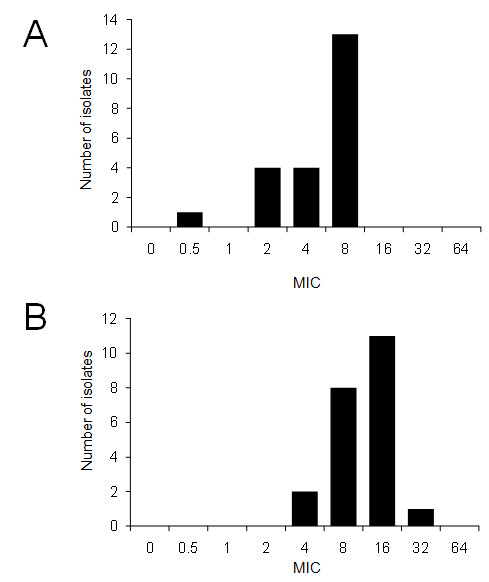
**Minimum inhibitory concentrations (MICs) of aminoglycosides in 22 strains of *Pseudomonas aeruginosa *isolated from chinchillas**. (A) Gentamicin (B) Amikacin.

**Figure 3:**
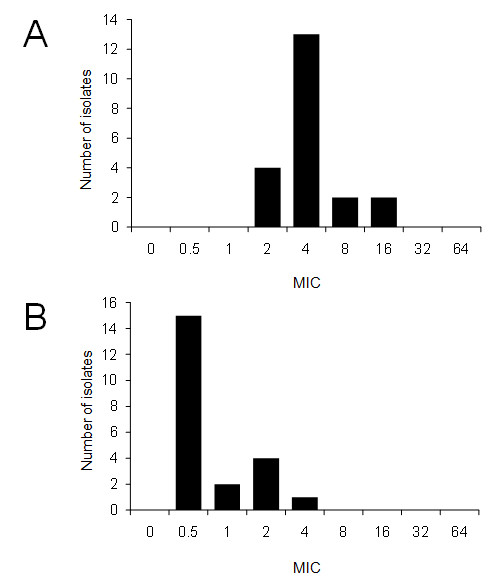
**Minimum inhibitory concentrations (MICs) of fluoroquinolones in 22 strains of *Pseudomonas aeruginosa *isolated from chinchillas**. (A) Enrofloxacin (B) Ciprofloxacin.

Figures 2A, B and 2C show the MICs for aminoglycosides, fluoroquinolones, and selected β-lactams, respectively. For the aminoglycoside susceptibility test, we selected gentamicin and amikacin (Figures 2). Approximately 60% of the isolates exhibited an MIC value greater than 8 μg/ml for gentamicin (Figure 2A). According to MIC interpretive standards for *P. aeruginosa *published by the National Committee Clinical and Laboratory Standards (NCCLS) [16], 9 and 13 isolates were susceptible and intermediate to gentamicin, respectively. Only one isolate was intermediate to amikacin according to the NCCL interpretive standards, and none of the isolates showed resistance to these 2 aminoglycosides (Figure 2B). With regard to fluoroquinolones, only one isolate showed an MIC value of 4 μg/ml for ciprofloxacin, while approximately 80% of the isolates showed an MIC value greater than 4 μg/ml for enrofloxacin (Figure 3A and 3B). According to the NCCL interpretive standards, one isolate was resistant to ciprofloxacin. Prior to sampling, 2 chinchillas bred as laboratory animals were treated with Baytril (enrofloxacin; Bayer, Leverkusen, Germany) for 6 days and Gentacin (gentamicin; Schering-plough animal health, Wim de Körverstraat, Netherlands) for 2 days for otitis media. The MICs of 2 isolates from these chinchillas were 2-4 μg/ml for enrofloxacin and 4-8 μg/ml for gentamicin, which may indicate that prior treatment led to the reduced susceptibility to these antibiotics. Except for 2 chinchillas, none of animals was treated with antibiotics.

**Figure 4:**
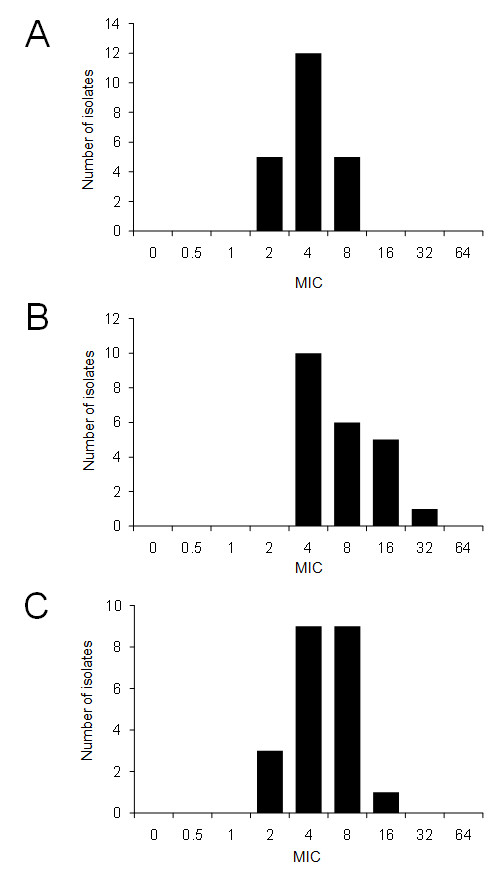
**Minimum inhibitory concentrations (MICs) of β-lactams in 22 strains of *Pseudomonas aeruginosa *isolated from chinchillas**. (A) Imipenem (B) Ceftazidime (C) Cefepime.

Further, we tested the β-lactam antibiotics that have been mainly used for human treatment, including imipenem and third and fourth generation cephalosporins (Figure 4A, B, and 4C). Five isolates that exhibited an MIC of 8 μg/ml were intermediate to imipenem, while 16 isolates showed an MIC value less than 4 μg/ml and were considered susceptible to imipenem according to the NCCL interpretive standards. With regard to cephalosporins, approximately 80% of the isolates were confirmed to be susceptible to both ceftazidime and cefepime; however, 6 and 1 isolates exhibited an MIC value greater than 16 μg/ml for ceftazidime and cefepime, respectively. For ceftazidime, these 6 isolates were revealed to be intermediate according to the NCCL interpretive standards. These results indicate that the isolates from chinchillas had a reduced susceptibility to the latter antibiotics used for humans, but not to those used for animals.

### Polymerase chain reaction (PCR) assay and sequencing

All isolates underwent PCR amplification to confirm the existence of genes encoding various types of ESBLs in *P. aeruginosa *wild strains [3,17]. None of the isolates were found to contain ESBLs by using primer pairs for OXA, TEM, SHV, PER, CTX group 2, CTX group 9 and VEB-type ESBLs. By using primer pairs GES and CTX group1, however, visible bands near the location of expected size were observed in 9 and 2 isolates, respectively (data not shown). To examine sequencing accuracy, TA cloning was performed. By using the GES primer pair, all the inserts that were sequenced were partially identified by blastx search as the ATP-binding cassette (ABC) transporter permease found in various strains of *P. aeruginosa*, while none of the inserts were found to contain the gene encoding the GES-type β-lactamase. By using the CTX group 1 primer-pair, none of the inserts were found by blastx search to contain the gene encoding the CTX-type β-lactamase. However, all the inserts from 2 PCR amplicons were highly similar to the gene encoding the putative PilL protein found in *P. aeruginosa *(accession no. ABR13432), with 82% coverage and 97% maximum identity by blastx search. Further, all the inserts were also similar to the gene encoding the type IV B pilus protein in *P. aeruginosa *(accession no. YP_792920), with 81% coverage and 95% maximum identity. Both the PilL and type IV pilus protein were identified in a novel pathogenicity island (PI) in highly virulent clinical isolates, including strain PSE9 and PA14 [18-20]. Although the CTX group 1 primer pair may be annealed with a similar sequence but not the targeting gene, the 2 isolates were confirmed to harbour the gene *pilL*. Although we attempted to isolate plasmid from these 2 strains, none of them was confirmed to harbour plasmid. These results also indicate that the 2 isolates containing the gene similar to *pilL *possibly also possess a PI in their genomes. The two chinchillas that were infected with the *P. aeruginosa *isolates containing the gene similar to *pilL *were bred as laboratory animals and 2 isolates were found to be susceptible to all tested antibiotics.

Although we also conducted tests to confirm the production of metallo-β-lactamase (MBL) in all isolates, no isolates were found to produce MBL. Compared with the serotype and the PFGE type, there was no correlation between typing and the susceptibility to antibiotics. Although the genes coding for ESBLs were not detected in chinchilla isolates using PCR assay and sequencing, the 2 isolates containing the gene *pilL *may be highly virulent strains of *P. aeruginosa*.

## Discussion

In this study, all the chinchillas used for *P. aeruginosa *isolation showed no clinical signs of *P. aeruginosa *infection or other bacterial infections, with the exception of 2 laboratory chinchillas with otitis media. *P. aeruginosa *was isolated from 30.4% of pet chinchillas and 47.7% of laboratory chinchillas. In total, *P. aeruginosa *was isolated at a rate of 41.8% in seemingly healthy chinchillas. Although otitis media caused by *P. aeruginosa *and their clinical signs have been reported in many previous studies in chinchillas [9-12], there may be a latent period explaining the lack of such symptoms in the infected animals included in our study. Further, clinical signs of *P. aeruginosa *infection may be developed under immunosuppressive conditions in host animals. Many cases of *P. aeruginosa *infection in chinchillas have been considered to be caused by environmental contaminations such as feed, water and flooring materials. In addition to these contaminations, transient infections may result in silent chronic infections in chinchillas due to coprophagia [21].

Although no evidence was found that the isolates from chinchillas were heterogeneous, serotype and PFGE analyses discriminated the isolates into 5 serotypes and 7 PFGE types, respectively. These results suggest that the isolates diversified despite limited numbers. We used slide agglutination for the determination of serotype as a minor modification of Homma's method [22]. In early epidemiological studies, *P. aeruginosa *isolates from both human and laboratory rodents were reportedly predominantly serotype G, as determined by slide agglutination [23,24]. Although serotype G was the second most dominant serotype among the isolates in our study, the isolates belonging to serotype G were further discriminated into 3 independent PFGE types. Serotype and PFGE type reportedly cross-react with each other using slide agglutination; however, serotype G determined by Homma's method was also divided into 2-4 serotypes by other agglutination methods [23]. Thus, there may be no homogeneity in the degree of DNA restriction analysis among these serotypes.

In this study, we determined MICs for 3 different spectrums of antibiotics, including aminoglycosides, fluoroquinolones, and β-lactams. Prior to the surveillance, all the chinchillas bred as both pets and laboratory animals were confirmed to be receiving no antibiotics, with the exception of 2 laboratory chinchillas. These 2 laboratory chinchillas were treated with enrofloxacin and gentamicin. Although 2 isolates from these animals were considered to be susceptible to enrofloxacin, one of the isolates showed intermediate to gentamicin. These results suggested that the treatment of the antibiotics was led to the reduced susceptibility of the isolate. In laboratory rodents, gentamicin and enrofloxacin have been generally used for the treatment of infectious diseases presumably caused by *P. aeruginosa *or another Gram-negative bacterium [10,25,26], while fluoroquinolones, carbapenems, and cephalosporins, such as ciprofloxacin, imipenem, and cefepime, may not be used for any bacterial infection in laboratory rodents in Japan and are strictly reserved for human use. In our study, no anamneses were found and none of antibiotics were administered in pet chinchillas. Thus, the isolates with reduced susceptibilities to ciprofloxacin, imipenem, and cephalosporins were considered to be transmitted by humans and the environment.

Recently, outbreaks of *P. aeruginosa *infections resistant to various antibiotics have reportedly increased. Of these infections, multidrug-resistant *P. aeruginosa *(MDRP), including EBSL- and MBL-producing *P. aeruginosa *causing nosocomial infections, has become a serious problem [27-30]. Therefore, the isolates from the chinchillas included in our study were further used for the determination of susceptibility to the antibiotics to which MDRP is resistant. From the antibiotic susceptibility tests, we determined that no isolates exhibited high MIC values for aminoglycosides, fluoroquinolones, or β-lactams. In particular, none of the isolates showed an MIC value greater than 16 μg/ml for imipenem and none revealed positive results on MBL tests, indicating that the isolates were not MDRP or MBL-producing *P. aeruginosa*. However, one isolate exhibited an MIC value greater than 32 μg/ml for ceftazidime, suggesting that this isolate may have been an ESBL-producing *P. aeruginosa *strain.

In this study, the gene encoding ESBLs could not be identified in *P. aeruginosa *isolates using previously reported primers (Additional file 2). However, the isolates that showed high MICs values for third and fourth generation cephalosporins were considered to have the genetic background for low susceptibility to these antibiotics. These genetic elements should be clarified in future studies. Although the gene encoding ESBLs could not be detected in the isolates using the primer pair CTX group 1, TA cloning identified one of the genes involved in pillin biogenesis. The partially identified gene was similar to the *pilL *gene that has been reported to be located in the PI of a virulent strain of *P. aeruginosa *[20]. The PI we observed was also homologous to a previously identified *P. aeruginosa *PI (PAPI-1) that was only found in strain PA14, which is highly virulent for animals and plants [18,19]. The genes encoding PilL and type IV B pilus protein were not identified in less virulent strains of *P. aeruginosa*, such as strain PAO1, but only in highly virulent clinical isolates, including strain PSE9 and PA14. Both proteins were previously found to be related to type IV pillin in the newly discovered PI [19,20]. Type IV pillin has been shown to be one of the most important factors for adherence to and invasion of host cells during infection [31]. For *P. aeruginosa*, the existence of the PI, such as PAPI-1, is considered to be closely related to virulence and pathogenicity for host animals. Thus, monitoring *P. aeruginosa *using genetic information, including the existence of a PI and antibiotic resistance, may help prevent the propagation of the pathogen.

## Conclusions

In this study, *P. aeruginosa *was isolated at high rates from pet and laboratory chinchillas. Typing analysis showed that the isolates were diversified, and the antibiotic susceptibility test showed low susceptibility levels in several isolates. Although we could not identify the gene encoding ESBLs, the isolates were found to contain the gene *pilL *that was previously identified only in highly virulent strains of *P. aeruginosa*. Thus, it is necessary to take into account the risk of infection from pets or laboratory chinchillas to humans. Because of rapid disease progression and high mortality rates often seen in chinchillas [10], maintaining a clean environment and monitoring should be performed to help prevent *P. aeruginosa *infection.

## Methods

### Isolation and identification of *P. aeruginosa *from chinchillas

The isolation of *P. aeruginosa *was carried in a total of 67 chinchillas, including 23 pets and 44 from 2 breeding facilities for use as laboratory animals. Chinchillas that were used for the isolation of *P. aeruginosa *were all healthy and had no clinical signs, but 1 chinchilla with otitis media was excluded. Further, 2 chinchillas bred as laboratory animals were treated with Baytril for 6 days and Gentacin for 2 days due to otitis media. These 2 laboratory chinchillas recovered completely from otitis media prior to sampling. Oral cavity, faeces, and individual water bottle swabs were collected from each chinchilla and diluted with sterilized saline. The samples were spread onto nalidixic acid cetrimide agar (Nissui, Tokyo, Japan) and incubated at 37°C for 48 h. Isolates were identified on the basis of colony pigment, Gram staining, and catalase activity. Subsequently, the isolates were identified as *P. aeruginosa *using Quick ID GN"Nissui" (Nissui). The serotype of *P. aeruginosa *isolates was determined with a slide agglutination test kit (Denka Seiken, Tokyo, Japan). Isolates that did not react to any antisera were determined to belong to a nontypeable strain. The glycerol stock of isolates was kept at -80°C until analysis. After this time, 6 isolates could not be revived, and therefore, a total of 22 isolates were used for the analyses. To ensure the accuracy of the identification procedures, 2 reference strains of *P. aeruginosa *ATCC 27853 (American Type Culture Collection, Manassas, VA, USA) and IID 1130 (The Institute of Medical Science, The University of Tokyo, Tokyo, Japan) were used in comparative analyses. Chinchillas were treated in accordance with the provisions for animal welfare of The Nippon Veterinary and Life Science University (formerly The Nippon Veterinary and Animal Science University), which follows the Guidelines for Animal Experimentation issued by the Japanese Association for Laboratory Animal Science.

### Pulsed-field gel electrophoresis analysis

PFGE was carried out as described previously [32,33]. Briefly, all isolates, which were grown in nutrient broth overnight at 37°C, were centrifuged at 10,000 *g *for 2 min. The pellet was resuspended and mixed with 1% agarose. Lysis was performed at 37°C for 1 h, followed by proteinase K digestion at 50°C for 16 h. After 4 washing steps, all plugs were digested with *Spe*I for 16 h at 25°C. After *Spe*I digestion, the fragments were separated by electrophoresis in 1% agarose gels with a CHEF-DR apparatus (Bio-Rad, CA, USA) for 18 h, with the switch times ranging from 5.3 s to 34.9 s and a field strength of 6 V/cm^2^. Lambda ladders were applied as molecular size makers (Bio-Rad). The gels were stained for 20 min with ethidium bromide and destained for 20 min thereafter. After being photographed under UV, fragment patterns were compared visually and cluster analysis by the unweighted-pair group method with arithmetic averages was performed using NTSYSpc (Exeter Software, NY, USA), as described previously [34].

### Antibiotic susceptibility test

MICs were defined as the lowest concentration of antibiotics that showed no visible growth after 24 h of incubation at 35°C. The MICs of antibiotics for all isolates were measured using a broth microdilution technique described by the Clinical and Laboratory Standards Institute [16]. Antimicrobials were obtained from the following sources: gentamicin (ICN Biomedicals, Aurora, Ohio), amikacin (Bristol Laboratories, Belleville, Ontario, Canada), imipenem (Merck, NJ, USA), ceftazidime (LKT Laboratories, MN, USA), cefepime (Bristol-Myers Squibb, NY, USA), enrofloxacin (MP Biomedicals, OH, USA), and ciprofloxacin (LKT Laboratories). All antimicrobials were thawed and diluted to the desired concentrations with cation-adjusted Mueller-Hinton II broth (BD, MD, USA) prior to each susceptibility test. Each test was repeated at least 3 times. All isolates were assessed for the production of MBLs using metallo-β-lactamase SMA "Eiken" (Eiken, Tokyo, Japan). To verify the susceptibility of isolates, the MIC values of aminoglycosides, ciprofloxacin, imipenem, and ceftazidime were compared with the description of the NCCL interpretive standards [16].

### DNA manipulation

We used PCR techniques to detect the gene encoding various types of ESBLs found in *P. aeruginosa *using the methods of Jiang et al. [17] and Weldhagen [8]. In brief, PCR was used to detect the gene encoding β-lactamases, OXA-10, TEM, SHV, PER-1, CTX group 1, CTX group 2, CTX group 9, VEB-1and GES (Additional file 2). The amplicons were confirmed by agarose gel electrophoresis, and visualized bands were subsequently purified with Suprec PCR (Takara bio, Shiga, Japan). The purified PCR products were ligated with T-vector, pTAC-1 using a TA PCR cloning kit (Biodynamics laboratory, Tokyo, Japan), and the resulting plasmids transformed in *Escherichia coli *DH5α were spread on Luria-Bertani medium supplemented with 100 μg/ml ampicillin. The colonies were randomly picked up and screened directly for inserts by performing colony PCR with primers M13-f and M13-r (Additional file 2). The PCR products were purified and cycle sequencing reaction was conducted using the BigDye terminator cycle sequencing kit (Applied Biosystems). The products were then analyzed using an ABI 310 DNA sequencer (Applied Biosystems). The closest matches of the sequences were identified by blastx search. For the several isolates, existence of plasmid was confirmed to isolate plasmid DNA using a Miniprep DNA purification kit (Takara). The results were confirmed on agarose gels under UV.

### Nucleic acid accession numbers

The nucleotide sequences of partial genes encoding ABC transporter permease (G-1) and PilL protein (C-1) were deposited in GenBank through DNA Data Bank of Japan (DDBJ), and the accession numbers assigned were AB591379 and AB591380, respectively.

## Authors' contributions

NA performed the survey of *P. aeruginosa *in chinchillas in 2002-2004. YH developed the study, including PFGE analysis and antibiotic susceptibility test. HS performed the PCR assay and sequencing, and coordinated the study. IH and TM supported antibiotic susceptibility of the isolates. EK, HK and HA supported and supervised the study. All authors read and approved the final manuscript.

## Supplementary Material

Additional file 1 **Pulsed-field gel electrophoresis binding patterns of selected reference strains and wild-type strains of *Pseudomonas aeruginosa *after *Spe*I digestion of total DNA**. Lanes: M, Bacteriophage lambda ladder DNA; 1, MA8; 2, MA6; 3, N3; 4, N2; 5, N4; 6, A1; 7, IID 1130; 8, MA4; 9, ATCC 27853. Click here for file

Additional file 2 **Primers used for polymerase chain reaction and sequencing**. Nucleotide sequences of primers used for PCR, TA cloning and sequencing were described. ESBL = extended-spectrum β-lactamase. Click here for file
