# Supplementary material for: Prevalence and analysis of Pseudomonas aeruginosa in chinchillas
Source: BMC Vet Res. 2010 Nov 17;6:52. doi: 10.1186/1746-6148-6-52 (PMC2994850; doi:10.1186/1746-6148-6-52)
Supplement: Additional file 1 — Pulsed-field gel electrophoresis binding patterns of selected reference strains and wild-type strains of Pseudomonas aeruginosa after SpeI digestion of total DNA. Lanes: M, Bacteriophage lambda ladder DNA; 1, MA8; 2, MA6; 3, N3; 4, N2; 5, N4; 6, A1; 7, IID 1130; 8, MA4; 9, ATCC 27853. [file 1746-6148-6-52-S1.PDF]

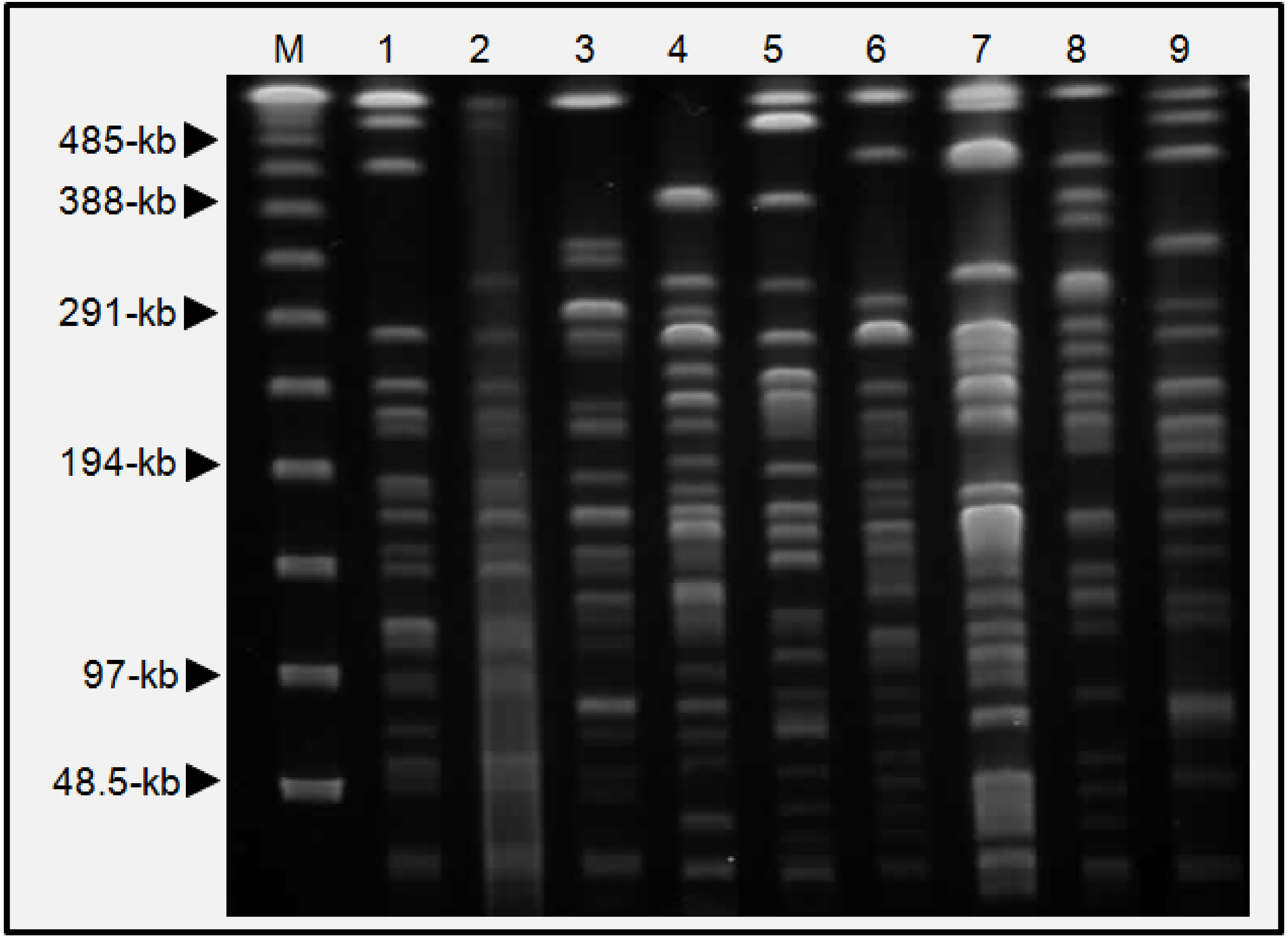

Additional file 1. Pulsed-field gel electrophoresis binding patterns of selected reference strains and wild-type strains of *Pseudomonas aeruginosa* after *Spe*I digestion of total DNA. Lanes: M, Bacteriophage lambda ladder DNA; 1, MA8; 2, MA6; 3, N3; 4, N2; 5, N4; 6, A1; 7, IID 1130; 8, MA4; 9, ATCC 27853.
