# Supplementary material for: Prevalence and analysis of Pseudomonas aeruginosa in chinchillas
Source: BMC Vet Res. 2010 Nov 17;6:52. doi: 10.1186/1746-6148-6-52 (PMC2994850; doi:10.1186/1746-6148-6-52)
Supplement: Additional file 2 — Primers used for polymerase chain reaction and sequencing. Nucleotide sequences of primers used for PCR, TA cloning and sequencing were described. ESBL = extended-spectrum β-lactamase. [file 1746-6148-6-52-S2.PDF]

Additional file 2 – Primers used for polymerase chain reaction and sequencing

ESBL type	Primer name	Sequence (5'-3')	Product size (bp)
TEM	TEM-f	ATAAAATTCTTGAAGAC	1,075
	TEM-r	TTACCAATGCTTAATCA	
SHV	SHV-f	TGGTTATGCGTTATATTCGCC	867
	SHV-r	GCTTAGCGTTGCCAGTGCT	
CTX group 1	CTX3-f	GGTTAAAAAATCACTGCGTC	863
	CTX3-r	TTGGTGACGATTTTAGCCGC	
CTX group 2	CTX2-f	ATGATGACTCAGAGCATTCG	865
	CTX2-r	TGGGTTACGATTTTCGCCGC	
CTX group 9	CTX9-f	ATGGTGACAAAGAGAGTGCA	870
	CTX9-r	CCCTTCGGCGATGATTCTC	
OXA	OXA-f	GTCTTTCG(A)AGTACGGCATTA	699
	OXA-r	ATTTTCTTAGCGGCAACTTAC	
PER	PER-f	AATTTGGGCTTAGGGCAGAA	933
	PER-r	ATGAATGTCATTATAAAAGC	
VEB	VEB-f	CGACTTCCATTTCCCGATGC	642
	VEB-r	GGACTCTGCAACAAATACGC	
GES	GES-f	GTTTTGCAATGTGCTCAACG	371
	GES-r	TGCCATAGCAATAGGCGTAG	
M13	M13-f	GTTTTCCCAGTCACGAC	-
	M13-r	CAGGAAACAGCTATGAC	

ESBL = extended-spectrum β-lactamase
